# Immature Teratoma of Uterine Origin: A Case Report and Literature Review

**DOI:** 10.7759/cureus.87765

**Published:** 2025-07-12

**Authors:** Yoshiro Makino, Ken Nakayama, Yasushi Sasaki, Yasuo Ueda, Miki Morioka

**Affiliations:** 1 Department of Obstetrics and Gynecology, Showa Medical University Fujigaoka Hospital, Yokohama, JPN; 2 Department of Clinical Pathology, Showa Medical University Fujigaoka Hospital, Yokohama, JPN

**Keywords:** case reports, germ cell neoplasms, gliomatosis peritonei, teratoma, uterine neoplasms

## Abstract

Immature uterine teratoma is an extremely rare extragonadal germ cell tumour with only a small number of reported cases. Given its rarity, standardised treatment guidelines are unavailable, requiring an individualised management approach. Post-treatment assessment remains challenging, particularly in differentiating gliomatosis peritonei, a benign glial implant, from residual malignancy, as this can critically influence subsequent decisions regarding additional surgery or chemotherapy. A 44-year-old woman presented with prolonged genital bleeding and a vaginal mass that was initially suspected to be a prolapsing submucosal fibroid. Surgery confirmed a grade 3 immature teratoma with peritoneal dissemination and ovarian metastasis, necessitating four cycles of bleomycin, etoposide, and cisplatin chemotherapy. Post-treatment imaging suggested a residual tumour; however, a secondary laparotomy revealed gliomatosis peritonei. Treatment decisions were guided by a comprehensive analysis of International Germ Cell Consensus Classification (IGCCC) and modified IGCCC risk stratifications, existing guidelines for gonadal and extragonadal germ cell tumours, and a review of past cases. Although limited by a small number of cases and clinical heterogeneity, findings from previous reports, including the present case, suggest a potential role for adjuvant chemotherapy in reducing recurrence, regardless of the tumour stage. Notably, even after chemotherapy, assessment continues to pose diagnostic difficulties in differentiating residual immature teratomas from gliomatosis peritonei, often requiring surgical confirmation. Optimal management of immature uterine teratomas has yet to be defined. This case underscores the importance of multimodal therapy and precise post-treatment evaluation for recurrence and residual disease. Further case accumulation and collaborative research are crucial to refine treatment strategies and improve outcomes.

## Introduction

Immature teratoma is a malignant subtype of germ cell tumour (GCT) that most commonly arises in the gonads. However, a subset of GCTs, known as extragonadal GCTs (EGCTs), can develop outside the gonads, typically along the midline of the body, most frequently in the mediastinum, retroperitoneum, or sacrococcygeal region [1,2]. Among extragonadal sites, the uterus is an exceedingly rare primary location of GCTs, with fewer than 20 published case reports of immature uterine teratomas. Owing to its extreme rarity, no standardised treatment guidelines exist, making optimal management strategies unclear, often requiring case-based decision-making. Additionally, gliomatosis peritonei (GP), a rare condition characterised by peritoneal dissemination of mature glial tissue, may occasionally accompany immature teratomas, most commonly those of ovarian origin [3], thereby complicating post-treatment evaluation. GP associated with immature uterine teratoma is an even more exceptional occurrence, with only two cases published to date [4,5]. Here, we report a rare case of an immature teratoma of uterine origin accompanied by GP and examine its initial treatment strategy and post-treatment assessment through a review of the existing literature. This report aims to contribute to the limited body of knowledge on immature uterine teratomas and underscore the diagnostic challenges associated with distinguishing GP from malignancy.

## Case presentation

A 44-year-old nulligravida woman consulted a gynaecologist for moderate genital bleeding persisting for a month following treatment with dienogest (1mg twice daily) for menorrhagia. She had regular monthly menstrual periods since her menarche at the age of 12. She had never been married and had no history of sexual intercourse. Her medical history included hypertension treated with amlodipine, essential thrombocythaemia managed with low-dose aspirin, neovascular glaucoma treated with orally administered kallidinogenase, and papillary thyroid cancer, which was incidentally detected and treated with total thyroidectomy and cervical lymphadenectomy three months prior to presentation, without adjuvant therapy. She is currently on levothyroxine replacement therapy. Regarding her family history, her father had died of prostate cancer and her mother had died of a soft tissue tumour identified as a solitary fibrous tumour. Her siblings had no notable medical history.

**Figure 1:**
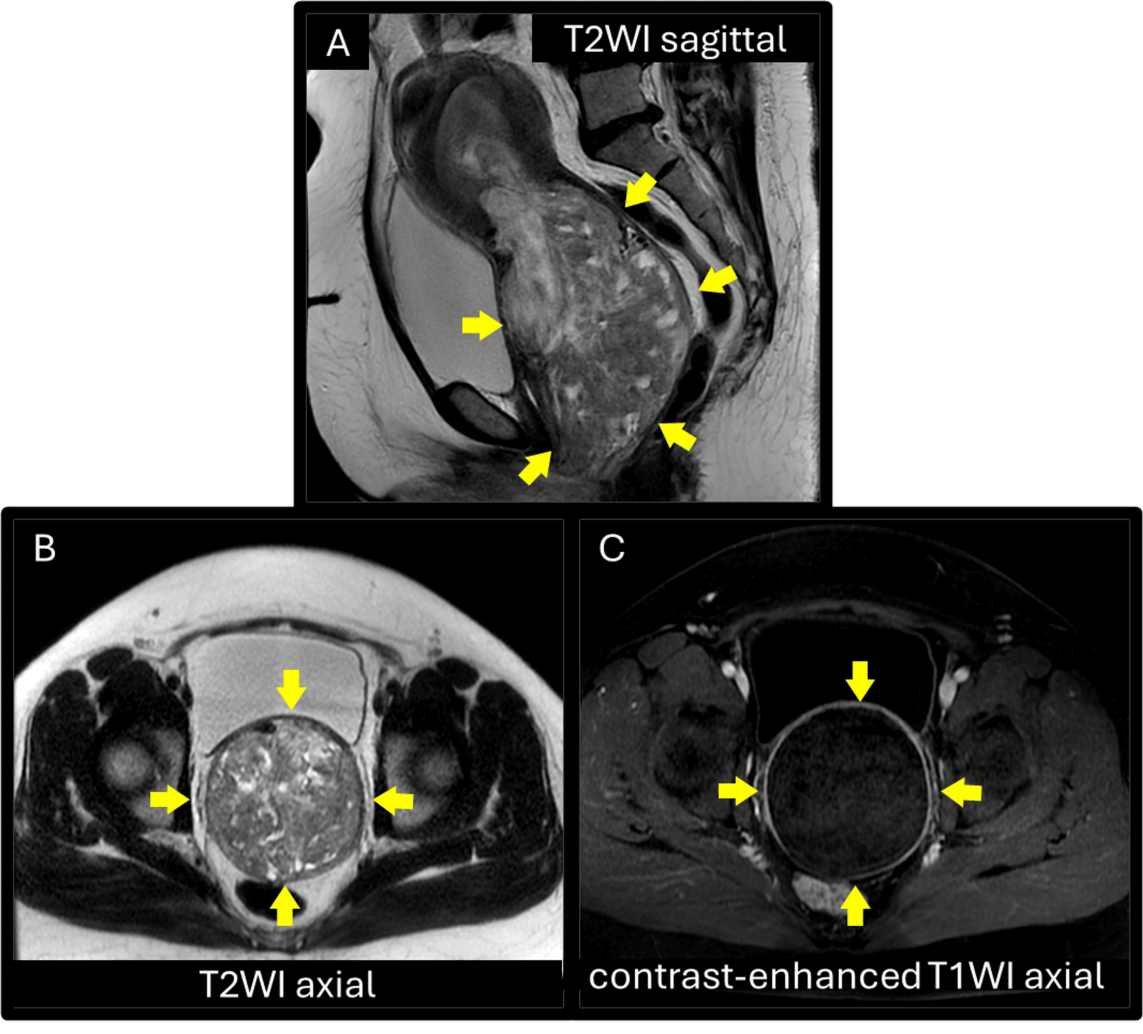
Pelvic magnetic resonance imaging of the patient at her first presentation prior to emergency hysteroscopic surgery. (A) T2-weighted mid-sagittal image showing the tumour with a heterogeneous signal arising from the uterine fundus and prolapsing into the vagina. (B) T2-weighted axial image and (C) contrast-enhanced T1-weighted axial image at the level of maximal diameter of the mass showing no contrast enhancement, indicating necrosis of the tumour. T2WI, T2-weighted image; T1W1, T1-weighted image.

At presentation to her gynaecologist, the patient was alert but febrile at 38.0 °C, with a blood pressure of 143/88 mmHg and pulse rate of 129 bpm. Physical examination revealed a mass measuring approximately 6 cm in diameter, protruding from the external cervical os into the vaginal cavity and presumed to be of uterine origin. The mass appeared dark purple in colour, soft in consistency, and suggestive of necrosis. Transabdominal ultrasonography and magnetic resonance imaging (MRI) revealed a mass measuring 14 × 9 cm originating from the uterine fundus and extending through the external cervical os into the vaginal cavity. The mass appeared heterogeneous on T2-weighted images and showed no enhancement on contrast-enhanced T1-weighted images (Figure 1).

Laboratory testing showed increased white blood cell count (19,260/µL; reference range: 3,900-9,800/µL), anaemia (Hb 9.0 g/dL; reference range: 11.3-15.2 g/dL), thrombocytosis (platelet count 545,000/µL; reference range: 130,000-369,000/µL), and elevated C-reactive protein (CRP 10.87 mg/dL; reference range: ≤0.14 mg/dL). The lactate dehydrogenase (LDH) was 183 U/L (reference range: 124-222 U/L), whereas other serum biochemical markers were within normal ranges. Tumour marker analysis showed CA125 at 125.2 U/mL (reference range: ≤35.0 U/mL), carcinoembryonic antigen (CEA) at 2.1 ng/mL (reference range: ≤5.0 ng/mL), and CA19-9 at < 2.0 U/mL (reference range: ≤37.0 U/mL).

Based on the findings from physical examination, imaging, and laboratory tests, the leading diagnosis at that time was an infected and necrotic prolapsing submucosal fibroid. Accordingly, owing to concerns of necrosis and infection, the patient was urgently taken to the theatre for definitive surgical management. Transvaginal mass removal and hysteroscopic resection of the mass stalk were performed to control infection and bleeding. The prolapsing mass was black-purple in colour, malodorous, and fragile, indicating extensive necrosis. The operation lasted 90 min, with an estimated blood loss of 170 mL, yielding a 370 g specimen. Postoperatively, the patient was administered tazobactam/piperacillin for seven days and discharged without evidence of retained infection.

Histopathological examination revealed a mixture of tissue elements, including cartilage, bone, hair follicles, fat, and cerebral cortex. Notably, immature neuroepithelial components were identified within one low-power field, confirming the diagnosis of a grade 1 immature teratoma.

**Figure 2:**
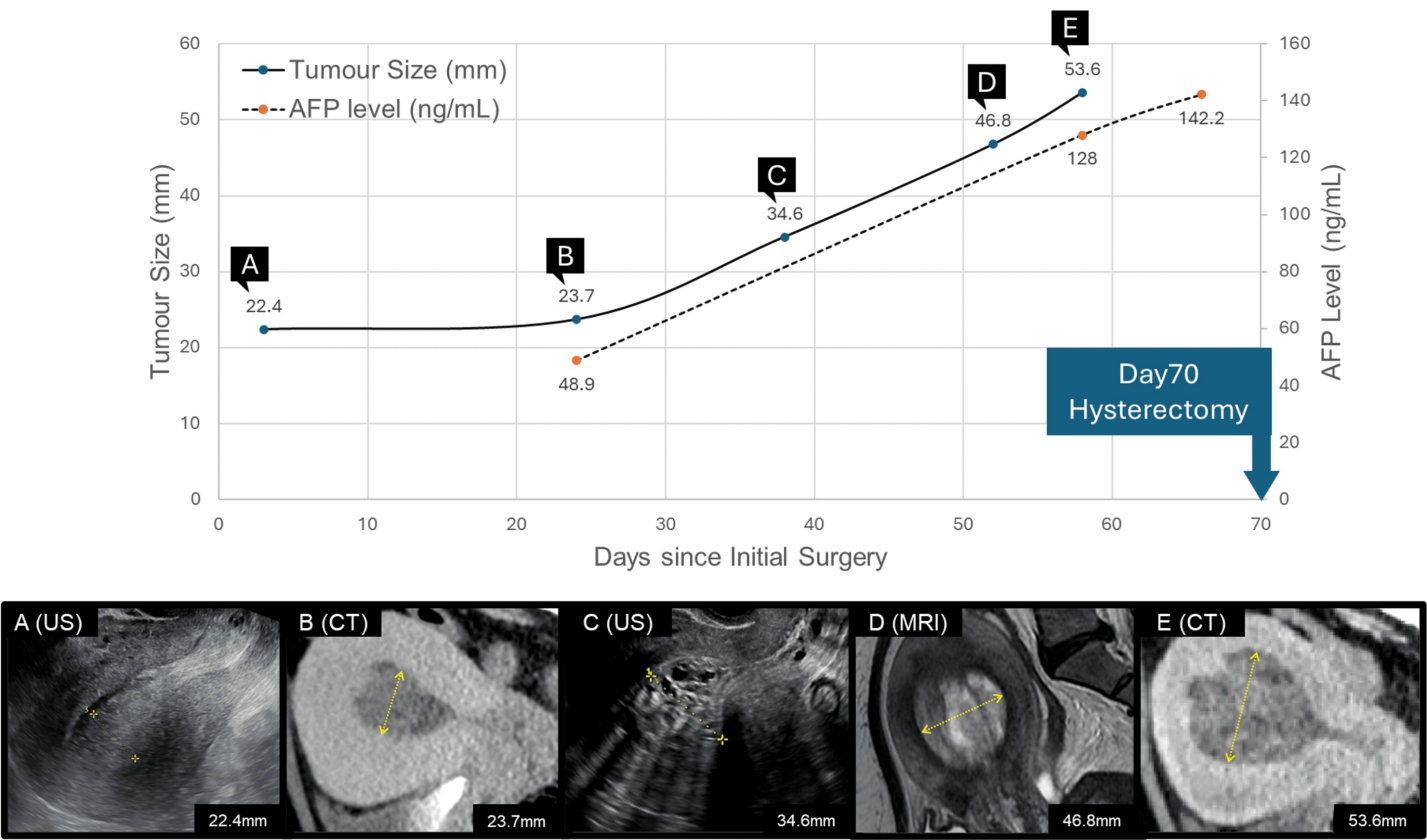
Chart of trends in the tumour size and AFP levels and serial pelvic imaging. Chart showing trends in tumour size and AFP levels, demonstrating tumour growth and rising AFP levels over time until hysterectomy. (A–E) Serial pelvic imaging demonstrating a growing intrauterine mass while waiting for elective surgery. AFP, alpha-fetoprotein; US, ultrasonography; CT, computed tomography; MRI, magnetic resonance imaging.

Systemic computed tomography (CT) following the infection-control surgery showed no evidence of metastasis. As the patient was placed at the end of the waiting list for elective surgery at the time of decision-making, radical surgery, including total hysterectomy, was initially scheduled four months after the initial transvaginal mass resection, resulting in a three-month delay. However, serial imaging studies, including ultrasonography, CT, and MRI, conducted during the waiting period revealed progressive enlargement of the intrauterine mass along with increasing alpha-fetoprotein (AFP) levels (Figure 2). Considering the risk of tumour progression, early surgical intervention was warranted.

Ultimately, total abdominal hysterectomy, bilateral salpingo-oophorectomy, and subtotal omentectomy were performed 70 days after the initial transvaginal surgery. The procedure took 5 h and 8 min, with a recorded blood loss of 1290 mL. The uterus was enlarged to the size of a grapefruit, with no abnormal findings on its serosal surface. However, an intrauterine mass was also observed. The fallopian tubes and ovaries were normal. Multiple disseminated nodules were observed in the vesicouterine and rectouterine pouches. Considering its widespread distribution, complete resection was not pursued. Although an extended surgical approach, including rectal resection, could achieve complete excision, this was not pursued because of the high chemosensitivity of immature teratomas and need for early initiation of adjuvant chemotherapy. Given these considerations, the potential disadvantages of excessive surgical morbidity were deemed to outweigh the benefits of complete resection.

**Figure 3:**
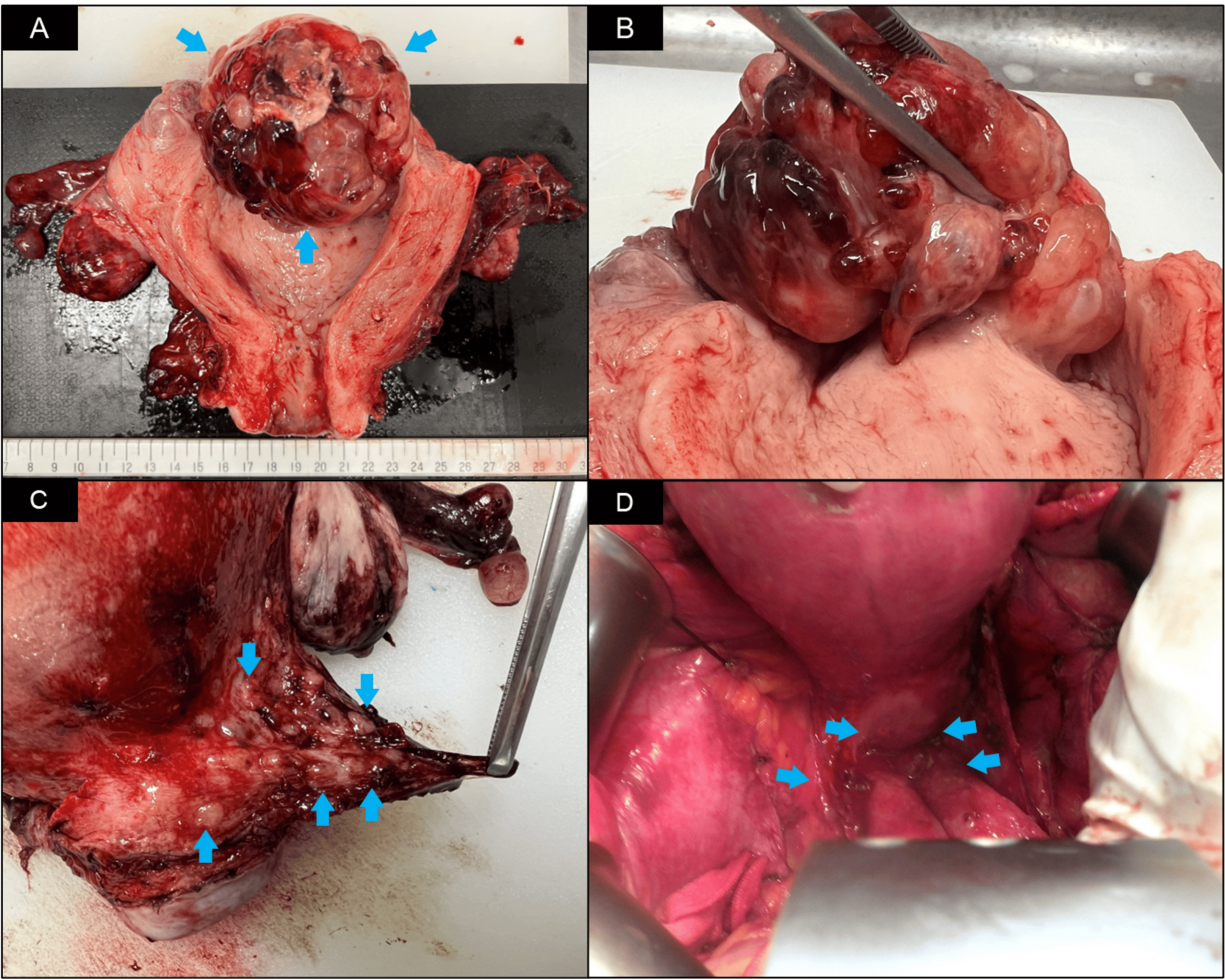
Macroscopic appearance of the intrauterine mass. (A) An intrauterine mass measuring 75 mm in diameter was identified. (B) The mass was pedunculated and attached to the fundal endometrium. (C, D) Multiple disseminated nodules were observed in the peritoneum of the rectouterine pouch.

**Figure 4:**
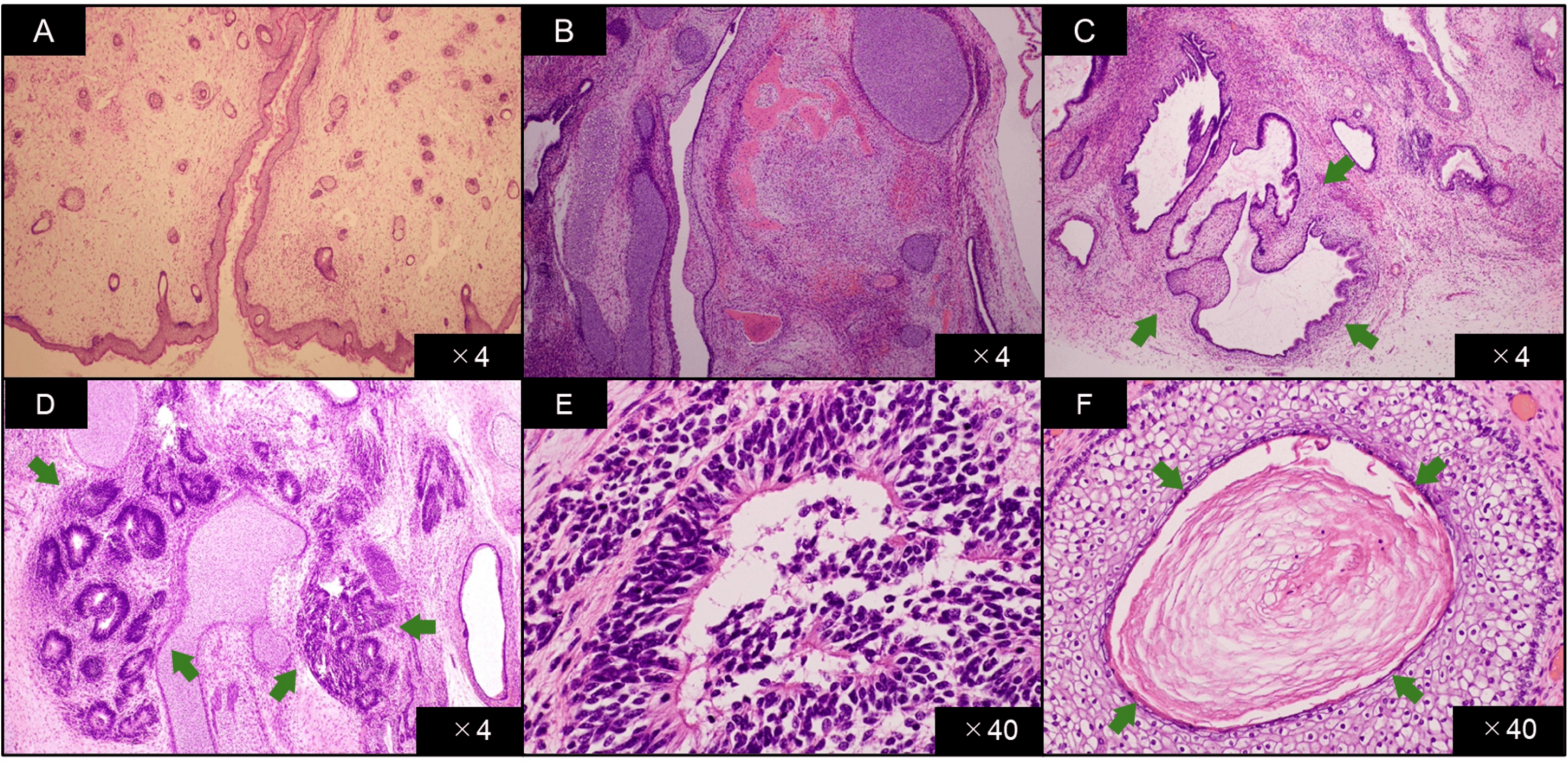
Microscopic examination of the tumour. (A) Mature tissue of skin and hair follicle, (B) mature tissue of bone and cartilage, (C) mature tissue of intestinal epithelium, (D) immature neural tube in low-power field, (E) immature neural tube in high-power field, and (F) metastatic immature squamous epithelium in the right ovary (high-power field).

Macroscopically, an exophytic intrauterine mass arising from the fundal endometrium was identified with no evidence of endometrial invasion (Figure 3). Microscopic examination revealed multiple tissue elements, including the skin, hair follicles, bone, cartilage, and intestine. The presence of immature neural tube elements and neuroepithelial components occupying five low-power fields confirmed the diagnosis of a grade 3 immature teratoma. Additionally, the peritoneally disseminated nodules contained the same tissue elements as the uterine masses. Further microscopic evaluation revealed mature cerebral tissue dissemination in the greater omentum and microscopic metastasis of immature squamous epithelium in the right ovary (Figure 4). The ascites cytology was negative for malignancy. Thus, the patient was diagnosed with a stage IV B immature teratoma of the uterine corpus.

**Figure 5:**
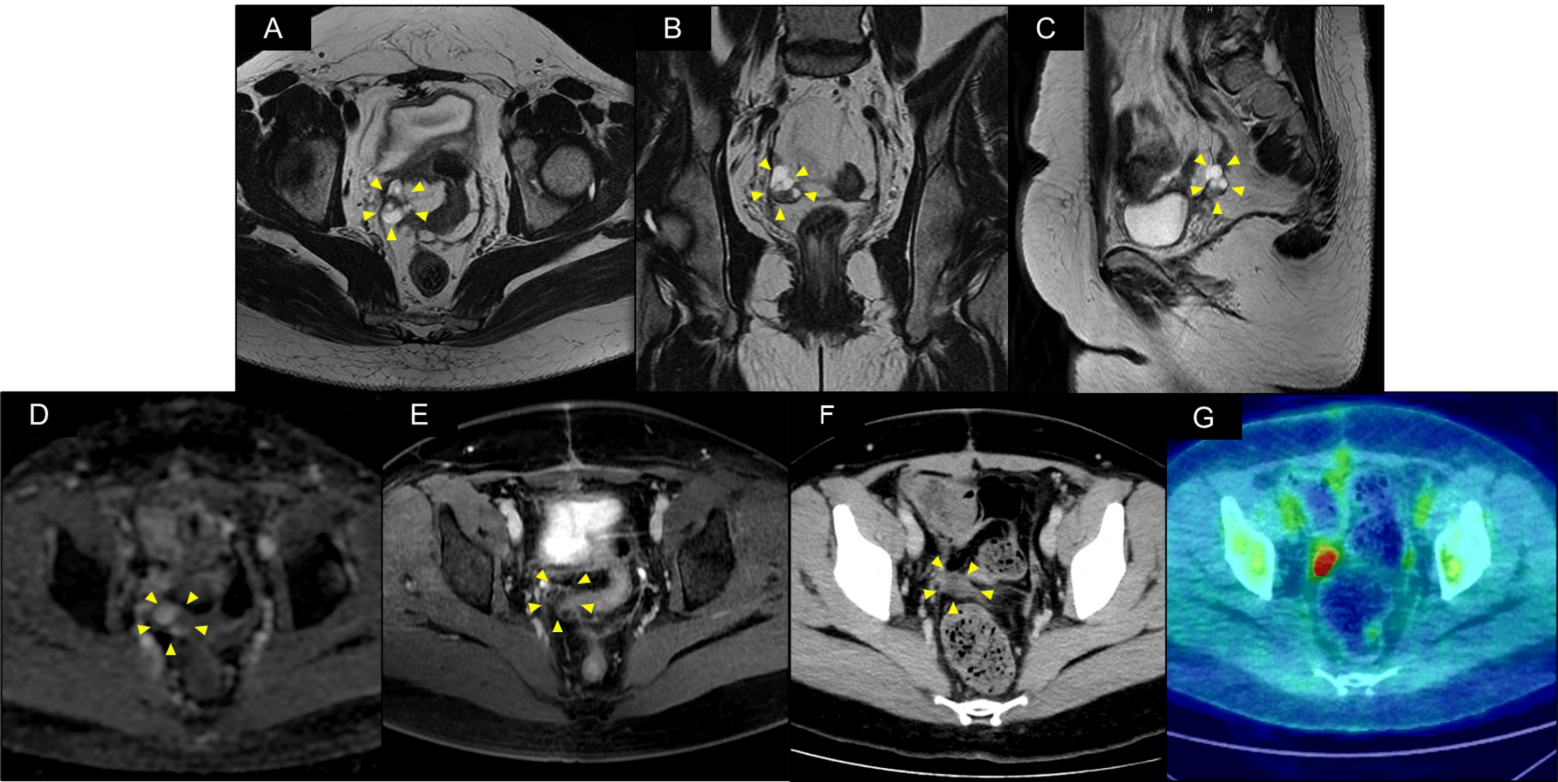
Pelvic imaging studies after four courses of postoperative chemotherapy. (A–C) Magnetic resonance imaging T2-weighted image axial, coronal, and sagittal cross-sections showing a mass with solid and cystic components on the right side of the rectum. (D) Apparent diffusion coefficient map demonstrating diffusion restriction in the solid component of the mass. (E, F) Contrast-enhanced T1-weighted image and contrast-enhanced computed tomography (CT) demonstrating delayed enhancement in the solid component of the mass. (G) Positron emission tomography/CT demonstrates ^18^F-fluorodeoxyglucose accumulation, with a maximum standardised uptake value of 25.65.

The patient subsequently received four cycles of combined chemotherapy with bleomycin, etoposide, and cisplatin (BEP). Post-treatment imaging, including CT, MRI, and positron emission tomography/computed tomography (PET/CT), was conducted to evaluate residual tumour presence. These studies revealed a 28 × 18 mm mass located in the pelvic peritoneum on the right side of the rectum. The mass exhibited both solid and cystic components with diffusion restriction and contrast enhancement on MRI. Additionally, PET/CT revealed significant 18F-fluorodeoxyglucose (FDG) accumulation, with a maximum standardised uptake value (SUVmax) of 25.65 (Figure 5). Tumour markers, including AFP and CA125, remained within normal limits despite abnormal imaging findings. The mass was suspected to be a residual tumour potentially unresponsive to chemotherapy. Owing to its solitary nature and the inability to determine its pathology through imaging alone, a secondary laparotomy for definitive diagnosis and complete excision was performed six months after the initial laparotomy. The surgery was completed within 3 h and 16 min, with a minimal blood loss of 40 mL.

Histopathological examination of the resected residual mass revealed that it primarily consisted of mature cerebral tissue, without any evidence of immature neural components. During the secondary laparotomy, a biopsy of the parietal peritoneum was performed, which revealed multiple nodules composed of mature nervous tissue. This led to a diagnosis of GP associated with an immature teratoma originating in the uterus.

The patient has been closely monitored and remains recurrence-free 15 months after the last surgery for GP and 17 months following the completion of chemotherapy for immature uterine teratoma.

## Discussion

GCTs most commonly originate in the gonads and predominantly affect males. However, 2-5% are classified as EGCTs. These tumours typically develop along the midline of the body, with the mediastinum (50-70%) and retroperitoneum (30-40%) being the most common sites. GCTs of uterine origin are exceedingly rare, with only 69 cases of all histological subtypes recorded in the SEER database from 1973 to 2007 [1,2].

The standard treatment approach for GCTs varies according to the primary location. In gonadal GCTs, surgical excision followed by chemotherapy, if necessary, is the preferred strategy, with risk stratification guiding postoperative management [2,6]. In contrast, mediastinal and retroperitoneal GCTs are primarily treated with chemotherapy, with resection reserved for residual tumours [2].

As no established guidelines exist for uterine GCTs owing to their extreme rarity, treatment approaches are often extrapolated from gonadal and extragonadal GCT management principles. The Japanese guidelines for testicular cancer recommend that EGCTs should be treated with chemotherapy based on International Germ Cell Consensus Classification (IGCCC) risk stratification, similar to testicular GCTs [2]. To address the need for a risk stratification model tailored to female GCTs, a modified IGCCC system was developed and validated for clinical use [7]. In this case, treatment was guided by principles applied to gonadal and EGCTs, incorporating IGCCC and modified IGCCC risk stratifications.

In the present case, the immature uterine teratoma was classified as having a poor prognosis based on the IGCCC risk stratification system. The modified IGCCC risk stratification for female GCTs also classified them into the poor prognosis group owing to ovarian metastasis. Consequently, the patient received four cycles of BEP combination chemotherapy, which is recommended in such cases [2,6].

A major diagnostic challenge in this case was determining whether the tumour originated in the uterus or the ovary. Whilst immature ovarian teratomas are relatively common, immature uterine teratomas are extremely rare. Microscopic examination of the ovarian lesion revealed only immature squamous epithelium, whereas the uterine mass contained tissues from all three germ layers. This histological difference suggested that the primary tumour was in the uterus, with the ovarian lesion representing a metastasis rather than a primary ovarian immature teratoma.

To better understand the best treatment options for immature uterine teratomas, we conducted a literature review of published case reports describing either immature teratomas or mixed GCTs with immature teratoma components originating from the uterus, including the cervix, corpus, and endometrium.

Our review identified 20 cases from 19 reports. Outcome data, available for 14 of these cases, are summarised in Table 1 [4,5,8-19]. Among these patients, seven of the eight who received adjuvant therapy remained disease-free, whereas one experienced recurrence and died. In contrast, recurrence occurred in two of the six patients who did not receive adjuvant therapy, both of whom subsequently died.

**Table 1:** List of reported cases of immature teratoma and mixed germ cell tumours with immature teratoma components of uterine origin, including the uterine corpus, cervix, and endometrium. IT, immature teratoma; EM Ca, endometrial carcinoma; Adeno Ca, adenocarcinoma; MGCT, mixed germ cell tumour; YST, yolk sac tumour; HG-ESS, high-grade endometrial stromal tumour; LN, lymph node; TAH, total abdominal hysterectomy; LSO, left salpingo-oophorectomy; BSO, bilateral salpingo-oophorectomy; PLA, pelvic lymphadenectomy; PALA, para-aortic lymphadenectomy; RH, radical hysterectomy; CT, chemotherapy; VAC, vincristine/dactinomycin/cyclophosphamide; BEP, bleomycin/etoposide/cisplatin; PTX, paclitaxel; IFO, ifosfamide.

Reference	Year of publication	Patient’s age (y)	Histological diagnosis	Site of metastasis	Local invasion	Surgery	Adjuvant therapy	Recurrence	Prognosis	Gliomatosis peritonei
Khorsandi et al. [8]	1981	27	IT	–	–	TAH + LSO	–	–	1-year survival	–
Ansah-Boateng et al. [4]	1985	37	IT with EM Ca	Ovary	–	TAH	Radiotherapy	–	2-year survival	+
Cortés et al. [9]	1990	13	IT	–	–	Tumorectomy	–	–	44-month survival	–
Iwanaga et al. [10]	1993	36	IT	–	–	TAH	CT (VAC)	–	5-year survival	–
Gomez-Lobo et al. [11]	2007	15	IT	–	N/A	Tumorectomy	CT (unspecified)	–	Chemotherapy in progress	–
Panesar et al. [12]	2007	38	IT	–	–	Conization	–	–	2-year survival	–
Newsom-Davis et al. [13]	2009	82	IT with Adeno Ca + myosarcoma	–	Myometrium	TAH + BSO, omentectomy	–	+	Died	–
Güzelmeri̇ç et al. [14]	2010	26	MGCT, mainly IT + YST	Ovary	Sigmoid colon, bladder	TAH + BSO, PLA + PALA, invaded organ resection	CT (BEP)	–	6-month postoperative survival	–
El Youbi et al. [15]	2013	56	IT	–	N/A	TAH	–	+	Died	–
Souza et al. [16]	2014	23	IT	–	Myometrium	TAH + BSO, PLA	CT (BEP)	–	12-month survival	–
Saffar et al. [17]	2017	23	IT with YST + carcinoid	Omentum, pelvic LN	Myometrium, ovarian surface	Modified RH, BSO+PLA, omentectomy	CT (BEP)	–	36-month postoperative survival	–
Stolnicu et al. [5]	2017	46	IT	–	–	Tumorectomy	–	–	12-year survival	+
Liu et al. [18]	2021	44	IT with HG-ESS	Pelvic LN, para-aortic LN	Myometrium, cervical stroma	TAH + BSO, PLA + PALA, omentectomy	CT (PTX/IFO)	+	Died	–
Zhao et al. [19]	2021	11	IT	–	–	Tumorectomy, ovarian cystectomy	CT (BEP)	-	2-year survival	–
Present case	2025	44	IT	Ovary, omentum	–	TAH + BSO, omentectomy	CT (BEP)	-	17-month survival	+

These findings suggest that adjuvant therapy after surgery, predominantly chemotherapy, reduces recurrence and improves survival. However, considering the small sample size and limited long-term follow-up data in several cases, further studies are required to confirm these observations.

Based on these literature findings and standard treatment principles for GCTs, we developed the following treatment strategy for this case. As the tumour was deemed to have originated from the uterus, a total hysterectomy was performed, as complete resection of the primary site was considered feasible. Additionally, given the previous reports of ovarian metastases and peritoneal dissemination in immature uterine teratomas, bilateral salpingo-oophorectomy and subtotal omentectomy were performed primarily for staging purposes. A thorough staging assessment is essential to determine the extent of the disease and guide subsequent treatment decisions.

Initially, total hysterectomy, bilateral salpingo-oophorectomy, and subtotal omentectomy were deemed sufficient for complete resection. However, intraoperative findings revealed extensive peritoneal dissemination, rendering complete cytoreduction through these procedures unachievable. Consequently, additional procedures, such as rectal resection, were considered to achieve complete excision. However, given the high chemosensitivity of immature teratomas and necessity for the early initiation of adjuvant chemotherapy, the potential disadvantages of excessive surgical morbidity outweigh the benefits of complete resection. Therefore, a more conservative surgical approach was selected to optimise the patient’s overall prognosis.

Adjuvant chemotherapy was administered in accordance with the guidelines for GCTs, which recommend systemic therapy for high-risk cases. Despite the lack of specific guidelines for immature uterine teratomas, the established survival benefit of chemotherapy in GCTs, particularly those of extragonadal origin, supports its use in our case.

The selected BEP regimen is the standard treatment for high-risk malignant GCTs. In this case, its use was justified based on the presence of ovarian metastasis and peritoneal involvement, which classified the tumour as having a poor prognosis according to the risk stratification systems.

Nine of the 15 patients, including our case, received adjuvant therapy, only one of whom experienced recurrence and succumbed to death. In contrast, two of the six patients who did not receive adjuvant therapy suffered recurrence and subsequently died. These observations further support the potential benefit of adjuvant chemotherapy in reducing recurrence and improving survival, although the limited sample size precludes definitive statistical conclusions.

Among the seven patients who received postoperative chemotherapy with documented regimen details, the sole patient who experienced recurrence and died was treated with combined paclitaxel and ifosfamide. Although the rationale for selecting this regimen was not specified, it may represent a modification of the paclitaxel, ifosfamide, and cisplatin (TIP) protocol, with the omission of cisplatin. However, the absence of cisplatin, a key agent in standard GCT regimens, including BEP or TIP, may have resulted in suboptimal efficacy. In contrast, all patients treated with standard GCT regimens, such as BEP and VAC (vincristine, dactinomycin, and cyclophosphamide), survived without recurrence. This trend suggests that using the standard GCT-specific chemotherapy is more effective for improving the outcomes for patients with immature uterine teratomas.

Importantly, the observed benefit of adjuvant therapy appeared independent of tumour stage. Among the five patients with documented extrauterine spread, including our own, all received either chemotherapy or radiotherapy, and only one patient (treated with paclitaxel and ifosfamide) experienced recurrence and died. Conversely, among the eight patients with disease explicitly confined to the uterus, five did not receive adjuvant therapy. One of these untreated patients experienced recurrence and died, whereas all three who received adjuvant chemotherapy survived. These findings suggest that postoperative therapy, particularly with standard GCT regimens, such as BEP, plays a more decisive role in prognosis than anatomical stage alone.

Regarding the role of hysterectomy, 10 of the 15 patients underwent surgical removal of the uterus, whereas five preserved it. Among the five uterine-confined cases that preserved the uterus, all patients survived. Notably, the single recurrence in the uterine-confined group occurred in a patient who had undergone hysterectomy but had not received adjuvant chemotherapy. This implies that a poor outcome was not associated with uterine preservation per se, but rather with omission of postoperative treatment.

Finally, in the two cases where the extent of local invasion was not specified, both patients had no documented metastasis. One patient underwent uterine preservation and was receiving unspecified chemotherapy at the time of reporting, with no recurrence noted; the other underwent hysterectomy without adjuvant therapy and subsequently died. These patterns further support the notion that prognosis in immature uterine teratomas may depend more on the use of appropriate adjuvant chemotherapy than on the surgical extent or disease stage alone.

Taken together, these observations underscore the need for an individualised approach to management, guided by tumour biology and therapeutic context.

Our approach highlights the necessity of individualised management for immature uterine teratomas, given the absence of standardised treatment protocols. Future case accumulation and systematic reviews are crucial for refining therapeutic strategies and evaluating the role of surgery and chemotherapy in improving patient outcomes.

Although surgical excision and adjuvant chemotherapy were completed as planned, post-treatment challenges remained in differentiating residual immature teratoma from GP, a distinction crucial for guiding further surgical decision-making and determining prognosis.

GP, a rare benign condition characterised by the peritoneal dissemination of mature glial tissue, is typically associated with immature teratomas. Definitive diagnosis of GP should be based on histopathological examination, as radiological findings are often inconclusive in differentiating GP from residual malignancy [3,20]. Histologically, GP is characterised by the presence of mature glial tissue, typically scattered along the peritoneal surfaces or within fibrous stroma. In typical cases, the glial cells lack cytological atypia and mitotic activity, supporting their benign nature. However, rare cases of malignant transformation have been reported [21,22], in which histological features, such as nuclear atypia and increased mitotic figures, may be observed. Immunohistochemical staining is usually positive for glial fibrillary acidic protein, confirming glial origin and tissue maturation [23,24]. Unlike residual immature teratomas, GP is resistant to chemotherapy and rarely exhibits malignant potential. Therefore, distinguishing GP from residual malignancy is crucial in post-treatment evaluation.

PET/CT findings can be misleading, as GP masses have been reported to exhibit FDG uptake owing to the high metabolic activity of mature cerebral tissue [25,26], mimicking malignant recurrence. This overlap complicates the differentiation and may lead to unnecessary interventions. Considering this diagnostic uncertainty, tumour markers, including AFP and CA125, remained within normal limits despite abnormal imaging findings, which, in retrospect, may have provided clues suggestive of GP rather than a persistent malignancy.

Although GP is benign, preoperative differentiation from residual immature teratomas remains challenging. In cases where preoperative findings strongly suggest GP, such as stable tumour markers and non-progressive imaging features, a 'watch and wait' strategy, involving close monitoring of tumour size changes before considering surgery, may be a viable alternative. However, the potential risk of disease progression if the mass is malignant necessitates careful consideration before opting for nonsurgical management.

Because GP typically does not require surgical excision unless symptomatic or suspected of rare malignant transformation, surgical intervention should be guided by clinical presentation, tumour size, and diagnostic necessity. In this case, owing to the uncertainty in the preoperative diagnosis and solitary nature of the lesion, complete resection was deemed appropriate. This case underscores the challenges of distinguishing GP from residual immature teratomas and the need for a multimodal approach that integrates imaging, tumour markers, and, when necessary, histopathological examination.

Given the lack of standardised treatment guidelines, therapeutic decisions for immature uterine teratomas must be individualised based on tumour biology, risk stratification, and the feasibility of surgical excision. Further case accumulation and collaborative research are essential to refine the diagnostic and therapeutic approaches for these rare tumours.

## Conclusions

GCTs originating in the uterus are extremely rare and optimal therapeutic strategies remain uncertain. Although immature uterine teratomas fall under the category of EGCTs, surgical resection is often more straightforward than resection of other extragonadal sites. Primary surgery followed by postoperative chemotherapy, guided by prognostic risk stratification and contextualised through a review of similar cases, resulted in remission in this case. However, distinguishing between GP and residual immature teratomas is a major post-treatment diagnostic challenge, as both demonstrate strong FDG uptake on PET/CT. Given this diagnostic uncertainty, repeated excisional surgeries may sometimes be unavoidable, raising the risk of over-treatment; thus, awareness of this condition is essential for clinical decision-making.

In the absence of standardised treatment guidelines, managing immature uterine teratomas requires an individualised approach that integrates tumour biology, risk classification, and surgical feasibility, in concert with a careful appraisal of previously reported cases. Including our present case, evidence suggests that adjuvant chemotherapy helps reduce recurrence regardless of the tumour stage, although the data remain limited. Tailored therapeutic planning is particularly relevant for cases accompanied by GP, where diagnostic uncertainty may influence surgical decision-making. Further case accumulation, systematic reviews, retrospective case series, and collaborative data analyses are instrumental in shaping future management strategies.
